# Functional Analyses of Three Targeted DNA Antimicrobial Peptides Derived from Goats

**DOI:** 10.3390/biom13101453

**Published:** 2023-09-27

**Authors:** Aili Wang, Mengying Zhou, Qian Chen, Hui Jin, Gaochi Xu, Ruiyin Guo, Jianmin Wang, Ren Lai

**Affiliations:** 1 Center for Evolution and Conservation Biology, Southern Marine Science and Engineering Guangdong Laboratory (Guangzhou), Guangzhou 511458, China; allie612@gmlab.ac.cn (A.W.);; 2 Suzhou Guangji Hospital, The Affiliated Guangji Hospital of Soochow University, Suzhou 215137, China; 3 Shandong Provincial Key Laboratory of Animal Biotechnology and Disease Control and Prevention, College of Animal Science and Veterinary Medicine, Shandong Agricultural University, Taian 271000, China; wangjm@sdau.edu.cn; 4 Key Laboratory of Bioactive Peptides of Yunnan Province, KIZ-CUHK Joint Laboratory of Bioresources and Molecular Research in Common Diseases, National Resource Center for Non-Human Primates, Kunming Primate Research Center, National Research Facility for Phenotypic & Genetic Analysis of Model Animals (Primate Facility), Sino-African Joint Research Center and Engineering Laboratory of Peptides, Kunming Institute of Zoology, Kunming 650107, China; 5 University of Chinese Academy of Sciences, Beijing 100049, China

**Keywords:** goat submandibular glands, antimicrobial peptides, antimicrobial, antioxidant, anti-inflammatory, DNA-binding

## Abstract

With the increase in drug-resistant bacteria, new antibacterial drugs have emerged as a prominent area of research and development. Antimicrobial peptides (AMPs), as innate immune agents, have garnered significant attention due to their potent, rapid, and broad-spectrum antibacterial activity. This study focused on investigating the functionality of three AMPs (CATH 1, CATH 2, and MAP34-B) derived from goat submandibular glands. Among these AMPs, CATH 2 and MAP34-B exhibited direct antibacterial activity against both Gram-negative and Gram-positive bacteria, primarily targeting the bacterial membrane. Additionally, these two AMPs were found to have the potential to induce reactive oxygen species (ROS) production in bacterial cells and interact with bacterial genome DNA, which may play a crucial role in their mechanisms of action. Furthermore, both CATH 1 and CATH 2 demonstrated significant antioxidant activity, and all three AMPs exhibited potential anti-inflammatory activity. Importantly, the cytotoxic activity of these AMPs against mammalian cells was found to be weak, and their hemolytic activity was extremely low. Overall, the characteristics of these three AMPs found in goat submandibular glands offer new insights for the study of host protection from an immunological perspective. They hold promise as potential candidates for the development of novel antibacterial agents, particularly in the context of combating drug-resistant bacteria.

## 1. Introduction

Cecropins, defensins, and magainins were successively discovered in the 1980s from the skin of silkworm moths, rabbit neutrophils, and African claw toads [1,2,3]. These peptides exhibit potent antimicrobial activity and are collectively referred to antimicrobial peptides (AMPs). Further research led to the discovery of a variety of AMPs from fungi, animals, and plants [4,5,6]. AMPs, which are found in many multicellular organisms, possess strong, rapid, and broad-spectrum antibacterial activity [7]. They play a crucial role in the host’s innate immune system and are also known as host-defense peptides (HDPs). In addition to their antimicrobial properties, AMPs exhibit multiple immunomodulatory activities and have a low rate of drug resistance development [7,8].

In addition to being a part of innate immunity, AMPs also exhibit broad-spectrum activity against pathogens. They achieve this through various mechanisms, including acting upon the cell membrane, forming transmembrane ion channels that disrupt its integrity and result in the release of cellular contents, ultimately leading to cell death, inhibiting membrane protein and DNA synthesis, causing single-stranded DNA breakage, interacting with DNA, producing hydrogen peroxide, inducing bacterial autolysis, and triggering apoptosis in eukaryotic cells [9].

Over 40 oral AMPs have been studied including cathelicidins, defensins, and histatins [10]. The peptides named cathelicidin due to their significant resemblance to the sequence of cathelin, an inhibitor of histonectin L (cathepsin L) that was initially purified from porcine neutrophils. Cathelin is a highly conserved region of approximately 100 amino acids, also known as the cathelin region. The conservation of this segment makes it an effective tool for identifying novel cathelicidins [11,12]. One of the most extensively researched cathelicidins is LL-37. Defensins are a class of antimicrobial peptides (AMPs) with a low molecular weight (4–5 kDa). Typically, defensins contain 6–8 cysteine residues and can form 3–4 pairs of intramolecular disulfide bonds. Depending on the specific locations of these disulfide bonds, defensins can be categorized into α-defensin, β-defensin, and θ-defensin [13]. α-defensins are primarily present in neutrophils, and the identified ones so far include hNP-1, hNP-2, hNP-3, hNP-4, hNP-5, and hNP-6. Among them, hNP-1, hNP-2, hNP-3, and hNP-4 are expressed in the oral cavity, while hNP-5 and hNP-6 are expressed in the human intestine. On the other hand, β-defensins, including hBD-1, hBD-2, hBD-3, hBD-4, hBD-5, and hBD-6, are mainly present in epithelial cells of various tissues and organs. Studies have demonstrated that only hBD-1, hBD-2, hBD-3, and hBD-4 are expressed in the oral cavity [14,15]. Histatins are a group of salivary proteins with a small molecular weight that are secreted by the parotid and submandibular glands. These proteins are rich in histidine (His), with a concentration of approximately 40–425 µg/mL in saliva. Histatins are small histidine-rich cationic peptides and typically consist of 7–38 amino acid residues [16]. Histatins have strong antifungal activity, and histatin 1, 3, and 5 are the main members found so far [17,18]. Additionally, histatin 2, 4, 5–12 are primarily derived from the proteolytic cleavage of histatin 1 and 3 [19,20,21]. The antifungal mechanism of histatins involves several stages, including binding to specific membranes, transmembrane operation, generation of reactive oxygen species (ROS) to hinder mitochondrial respiration, and mobilization of ions (K^+^ and Mg^2+^) into the cell, leading to cell death [22]. 

Ruminants, including cattle, horses, sheep, and deer, are known to be a valuable source of AMPs [23,24,25,26]. Five goat AMPs with antimicrobial activities have been studied, including ChBac5 [24], ChBac3.4 [6], mini-ChBac7.5Nbeta [27], mini-ChBac7.5Nalpha [27], and Gallocin D [28]. AMPs serve as a natural defense mechanism against microbial infections and play an important role in the response to inflammation. These peptides exhibit a wide range of antimicrobial properties and vary in size, composition, and mechanism of action. They are expressed in various tissues, including neutrophils, macrophages, and mucosal epithelial cells. Additionally, ruminants can be utilized as models for evaluating the effectiveness of AMPs, studying their wound-healing effects, and exploring their immunological properties [29].

In our previous transcriptome analyses [30], three significantly differentially expressed cathelicidin family AMPs were screened: *CATH 1*, *CATH 2* and *MAP34-B*. Analysis of the amino acid composition in their mature peptide sequences revealed a higher content of arginine in CATH 1, CATH 2, and MAP34-B, suggesting that these peptides may possess antibacterial activity due to their cationic nature. To further investigate their biological functions, we chemically synthesized the three peptides and conducted a systematic study of their biological activities.

## 2. Materials and Methods

### 2.1. Peptide Synthesis

The peptides were synthesized by GL Biochem Ltd. (Shanghai, China) using a peptide synthesizer. After synthesis, the peptides underwent purification and were then analyzed using HPLC and MALDI-TOF MS to confirm their purity (>98%). Physical and chemical parameters analysis was conducted using the ExPASy Bioinformatics website (http://www.expasy.org/tools/ (accessed on 21 March 2023)). The secondary structure of the peptides was predicted using the PEP-FOLD3 algorithm (http://bioserv.rpbs.univ-paris-diderot.fr/services/PEP-FOLD3 (accessed on 25 March 2023)). The SOPMA method was employed to determine the secondary structure content of the peptides (https://npsa-prabi.ibcp.fr/cgi-bin/npsa_automat.pl?page=npsa_sopma.html (accessed on 25 March 2023)).

### 2.2. Antimicrobial Assay

The antimicrobial activities of MAP34-B, CATH 1, and CATH 2 were evaluated using a two-fold broth microdilution method as described in the literature [31]. In this study, a total of 5 g-negative bacteria, 4 g-positive bacteria, and 1 fungus were utilized. The standard strains were stored in our lab while the clinical strains were collected from local hospitals. All microbes were incubated to exponential phase in Mueller–Hinton broth (MHB) at 37 °C and diluted to a concentration of 10^6^ CFU/mL with fresh MHB. Serial dilutions of MAP34-B, CATH 1, and CATH2 (50 µL) were prepared in a microtiter plate containing 96 wells and mixed with an equal volume of microbial inoculum. MIC values were determined as the lowest concentrations with no visible microbial growth after incubating plates at 37 °C for 18 h. Ampicillin and meropenem were used as positive controls for three conventional antibiotics.

### 2.3. Anti-Inflammatory Activity

Sodium thioglycolate (mercaptoacetate, 4 mL, 3%) was administered via intraperitoneal injection to C57BL/6 mice (6–8 weeks old), followed by sacrifice three days later. Immediately after, approximately 5 mL of precooled 1 × PBS was injected into the abdominal cavity of the mice without causing any damage to internal organs. The peritoneal cavity was gently rubbed for 5–10 min to collect the peritoneal fluid. The cells were then centrifuged at 1000 rpm for 5 min, discarding the supernatant, and resuspended in precooled medium (RPMI 1640) containing 10% fetal bovine serum. After thorough blowing and mixing, the cells were counted using a hemocytometer plate and diluted to a concentration of 10^5^ cells/mL with RPMI 1640 medium containing 10% fetal bovine serum. Two thousand macrophages were added to each well of sterile 96-well plates. After 3–5 h, the cells were washed three times with PBS to remove non-adherent cells. Then, RPMI 1640 medium supplemented with 2% serum and LPS (Sigma, Saint Louis, MO, USA) was added to the cell culture at a final concentration of 100 ng/mL, along with varying concentrations of peptide samples. PBS solution served as the negative control, and each group was replicated five times. Following a six-hour incubation at 37 °C, the supernatant was collected and IL-6 concentration was measured using an ELISA kit (eBiosciences, San Diego, CA, USA). The study was approved by the Institutional Review Board and Animal Care and Use Committee at Kunming Institute of Zoology (IACUC-RE-2022-08-007).

### 2.4. Bacterial Killing Kinetic Assay

This part of the assay aimed to evaluate the effectiveness of MAP34-B, CATH 1, and CATH 2 against bacteria by measuring the colony numbers on LB agar plates at different time points. The experiment followed the method described by Wang et al. [32]. *E. coli* CMCC44102 and *S. aureus* CMCC26003 were used as the test bacteria. The positive control was ampicillin, while sterilized ultrapure water served as the negative control. The test bacteria were cultured in MHB broth until they reached the logarithmic growth stage and were then diluted to a concentration of 1 × 10^6^ CFU/mL. The peptides were added to the bacteria at a final concentration of 5 × MIC and incubated at 37 °C for 0, 10, 30, 60, 180, and 360 min. At each time point, 50 μL aliquots were taken and diluted with fresh MHB broth. The diluted samples were then plated on LB agar plates, and the colony numbers were recorded after incubation. Three parallel tests were performed in each group, and the Mean ± SEM was calculated.

### 2.5. Cytoplasmic Membrane Permeabilization Assay

The permeability of the cytoplasmic membrane induced by peptides was investigated using two methods: propidium iodide (PI) permeation assay and calcein-AM permeation assay, following Wang et al.’s method [32]. *E. coli* CMCC44102 and *S. aureus* CMCC26003 were cultured in MHB until they reached the exponential phase. Then, they were washed and diluted to a concentration of 1 × 10^8^ CFU/mL using fresh MHB. A final concentration of 25 μg/mL of PI was added to the bacterial dilution. After a 30 min incubation in the absence of light at ambient temperature, bacterial dilutions (50 µL) were combined with MAP34-B, CATH 1, and CATH 2 at concentrations of 1×, 2× and 5 × MIC, respectively. The mixtures were then transferred to sterile black microtiter plates (96-well), and further incubated for an hour under dark conditions at room temperature. Fluorescence was measured using a Tecan Infinite M1000 PRO microplate reader (excitation: 535 nm; emission: 615 nm) from Tecan Switzerland. To calculate the percentage of cytoplasmic membrane permeability, 1% Triton X-100 (*v*/*v*) was used. The following equation was used to convert the results to a percentage of relative fluorescence intensity (RFI):

RFI (%) = (FI_peptide_ − FI_water_)/(FI_Triton X-100_ − FI_water_) × 100% (1)

In each group, three parallel tests were carried out. Mean ± SEM was calculated.

In comparison to the PI assay, the calcein-AM permeation assay involved the addition of calcein-AM (Yeasen, Shanghai, China) to the *S. aureus* dilution at a final concentration of 5 µM. The mixture was then incubated at 37 °C for 90 min. Bacterial suspensions and peptide samples were taken in 50 µL amounts to create peptide concentrations of 1 × MIC, 2 × MIC, and 5 × MIC, respectively. These samples were then incubated in sterile 96-well plates for 2 h at 37 °C. The fluorescence data were recorded using a Tecan Infinite (Tecan, Männedorf, Switzerland), with 1% Triton X-100 (*v*/*v*) being used as a positive control. The calculation equation used to determine the RFI (%) is as follows:

RFI (%) = (FC_peptide_ − FC_water_)/(FC_Triton X-100_ − FC_water_) × 100% (2)

FC_peptide_ represents the fluorescence value at a specific sample concentration. To ensure accuracy, three parallel experiments were conducted and the Mean ± SEM was calculated.

### 2.6. Detection of Bacterial Outer Membrane Permeability

The method used for detecting outer membrane permeability in Gram-negative bacteria was based on the protocol described by Zhang et al. [33]. *E. coli* CMCC44102 and *E. coli* ATCC25922 were cultured in MHB until they reached the exponential growth phase. Afterward, they were washed three times with HEPES buffer (pH 7.4, containing 5 mM glucose), and diluted to a concentration of 1 × 10^5^ CFU/mL. The fluorochrome N-phenyl-1-naphthylamine (NPN, sigma) was dissolved in dimethyl sulfoxide and added to the suspension at a final concentration of 10 µM. Subsequently, 100 µL of the suspension was added to each well of a 96-well plate, and incubated for 30 min at 37 °C. The background fluorescence was measured using a full-wavelength multifunctional microplate reader (Tecan, Switzerland) with excitation at 350 nm and emission at 420 nm. Peptides were added to each well at final concentrations of 1 × MIC, 2 × MIC, and 5 × MIC, respectively. The plates were then incubated for one hour at 37 °C. After incubation, the fluorescence values were measured using a full-wavelength multifunctional microplate reader (Tecan, Switzerland). Polymyxin B (0.1 mg/mL) was used as a positive control, representing 100% outer membrane permeability (F_100_). The results were converted to the percentage absorption of NPN using the following formula:

NPN_uptake_ (%) = (F_peptides_ − F_0_)/(F_100_ − F_0_) × 100% (3)

The fluorescence absorption values of the peptides at certain concentrations are represented by F_peptides_. F_0_ represents the initial fluorescence value of NPN without peptide addition, and F_100_ represents the fluorescence value of polymyxin B at a concentration of 0.1 mg/mL. Three parallel tests were conducted and the Mean ± SEM was calculated.

### 2.7. ABTS Radical Scavenging Assay

The test was conducted as described by Gião et al. [34]. The ABTS was dissolved in the PBS buffer to a final concentration of 2 mM to create the ABTS storage solution. This solution was mixed with a 70 mM potassium persulfate aqueous solution in a volume ratio of 250:1 to prepare the ABTS^+^ solution. The ABTS^+^ solution was kept at room temperature for approximately 16 h without exposure to light before use. Before the test, the ABTS^+^ solution was diluted with PBS to achieve an absorbance value of 0.80 ± 0.03 at 734 nm. A mixture of 2 μL peptide samples (2 mg/mL) and 48 μL of the aforementioned corrected ABTS^+^ solution was prepared and left at room temperature for 10 min. The absorbance value of the reaction solution was measured at 734 nm, with the solution medium of the sample used as a blank control. To calculate the percentage clearance rate of ABTS^+^, the results were converted as follows: A_B_ represents the absorption value of the blank control, and A_A_ represents the absorbance value of the sample. The ABTS^+^ clearance rate (%) was calculated using the formula (A_B_ − A_A_)/A_B_ × 100.

### 2.8. DPPH Radical Scavenging Assay

The DPPH radical scavenging assay was conducted following the methodology outlined by Feng et al. [35]. A 6 × 10^−5^ M solution of DPPH (Sigma, USA) was prepared in methanol. Then, 48 μL of DPPH solution was mixed with 2 μL peptide samples (2 mg/mL). The mixture was incubated at room temperature in the dark for 30 min, and the absorbance value at 517 nm was measured. Deionized water was used as a blank control. The percentage of DPPH scavenging by peptide sample was calculated according to the formula DPPH·clearance ratio % = (A_blank_ − A_peptide_) × 100/A_blank_.

### 2.9. Detection of Intracellular Reactive Oxygen Species (ROS)

The level of ROS induced by peptides in bacteria was assessed using the methods outlined by wang et al. [32]. *E. coli* CMCC44102 and *S. aureus* CMCC26003 were cultured to the exponential phase in MHB broth, washed thrice with PBS (pH 7.4), and subsequently diluted to a concentration of 5 × 10^8^ CFU/mL. The bacterial suspension was treated with 2′,7′-dichlorofluorescein diacetate (DCFH-DA, Beyotime, Nantong, China) at a concentration of 10 µM and incubated at 37 °C for 30 min for fluorescence-probing purposes. Then, 190 µL of probe-labeled bacterial suspension was added to a 96-well plate along with 10 µL peptide samples. After incubation at 37 °C for another 30 min, fluorescence was measured using a full-wavelength multifunctional microplate reader (Tecan, Switzerland) with an excitation wavelength of 488 nm and emission wavelength of 525 nm. Three parallel tests were performed, and Mean ± SEM values were calculated. Polymyxin B was used as a positive control.

### 2.10. Bacterial Genomic DNA Binding Assay 

A gel retardation assay was performed to investigate the binding of MAP34-B, CATH 1 and CATH 2 to bacterial genomic DNA. The genomic DNA was extracted from the bacteria *E. coli* ATCC25922 using a TIANamp Bacteria DNA Kit (TIANGEN, Beijing, China). Then, bacterial genomic DNA was incubated with serial dilutions of MAP34-B, CATH 1 and CATH 2 in a ratio of 1:10 at room temperature for 10 min at room temperature. Afterward, the mixtures were subjected to electrophoresis on a 1% agarose gel under the control of Polymyxin B.

### 2.11. Cytotoxic Assay

The cytotoxicity of MAP34-B, CATH 1 and CATH 2 against HepG2 cells, 4T1 cells and hacat cells was evaluated using the MTT method, following established protocols [36]. The concentrations of MAP34-B, CATH 1 and CATH 2 in the assay ranged from 25 to 200 μg/mL, in increments of 25 μg/mL.

### 2.12. Hemolytic Assay

The hemolysis of MAP34-B, CATH 1, and CATH 2 against mouse erythrocytes was evaluated using a previously described method [36]. Fresh mouse erythrocytes were washed three times with 0.9% saline and then incubated with different concentrations of MAP34-B, CATH 1, and CATH 2 (25, 50, 100, 150, and 200 μg/mL) at a temperature of 37 °C for 30 min. After centrifugation, the OD540 of the supernatant was measured to determine the level of hemolysis. A concentration of 1% Triton X-100 (*v*/*v*) was used as a positive control for complete hemolysis.

## 3. Results

### 3.1. Physicochemical Properties of AMPs

**Figure 1:**
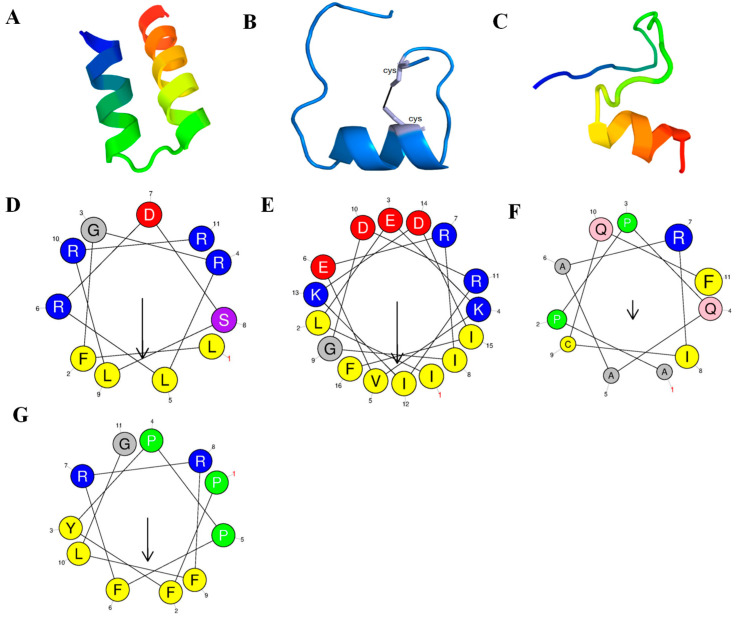
Secondary-structure determination of AMPs. (**A**–**C**) Secondary-structure prediction of MAP34-B, CATH 1, and CATH 2, the silver represents cysteine. (**D**,**E**) Helix-wheel diagram of MAP34-B, (**F**,**G**) Helix-wheel diagram of CATH 1 and CATH 2. Hydrophobic amino acids are shown in yellow and gray. Hydrophilic amino acids are shown in blue, purple and red.

The physicochemical properties of three highly expressed peptides in goat submandibular glands, namely MAP34-B, CATH 1, and CATH 2, are presented in Table 1. These peptides are all cationic and consist of mature sequences containing 34, 25, and 29 amino acids, with isoelectric points of 11.29, 11.54, and 12.54, respectively. Their theoretical molecular weights were 3955.63, 2951.59, and 3746.53 Da, and measured molecular masses were 3955.66, 2949.61, and 3746.56 Da, respectively. Figure 1 illustrates the predicted secondary structures of these peptides. MAP34-B exhibits an α helix structure at amino acid residues 2–12 and 17–32 at the N- and C-terminus, respectively, with the middle part forming random curls. CATH 1 and CATH 2 display an α-helical structure at the N-terminal amino acid residues 10–17 and 19–25, respectively, while the remaining amino acid residues are irregularly coiled.

### 3.2. Antimicrobial Activity

In this study, a total of 10 microorganisms were used, including 5 g-negative bacteria, 4 g-positive bacteria, and 1 fungus. The results presented in Table 2 indicate that CATH 1 did not exhibit direct antimicrobial activity against any of the tested strains. All the CATH 2, MAP34-B and the antibiotics showed no direct antimicrobial activities against the fungus. However, both CATH 2 and MAP34-B demonstrated broad-spectrum and potent antimicrobial activity, particularly against Gram-negative bacteria. MIC for MAP34-B ranged from 9.38 to 37.5 μg/mL, while for CATH 2 it ranged from 18.75 to 37.5 μg/mL.

### 3.3. Bacterial Killing Kinetic Assay

**Figure 2:**
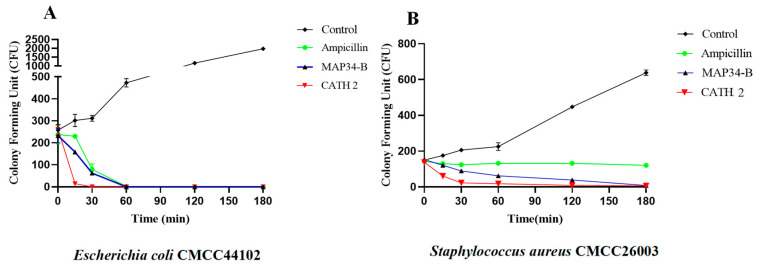
Bactericidal kinetics of CATH 2 and MAP34-B against *E. coli* CMCC44102 and *S. aureus* CMCC26003. (**A**) Bactericidal kinetics of CATH 2 and MAP34-B against *E. coli* CMCC44102, (**B**) Bactericidal kinetics of CATH 2 and MAP34-B against *S. aureus* CMCC26003.

The bactericidal rate of 5 × MIC CATH 2 and MAP34-B against *E. coli* CMCC44102 and *S. aureus* CMCC26003 is shown in Figure 2A,B. CATH 2 and MAP34-B were able to kill *E. coli* CMCC44102 within 30 min and 60 min. Similarly, they were able to kill *S. aureus* CMCC26003 within 180 min, while the positive control ampicillin took more than 180 min to kill *S. aureus*.

### 3.4. Cytoplasmic Membrane Permeability Test

**Figure 3:**
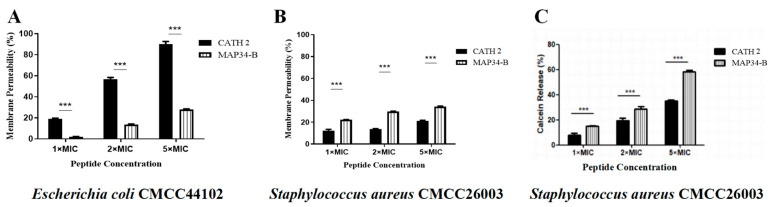
Cytoplasmic membrane permeability assay of CATH 2 and MAP34-B. (**A**,**B**) Fluorescent dye PI penetration in *S. aureus* CMCC26003 and *E. coli* CMCC44102 induced by CATH 2 and MAP34-B. (**C**) Fluorescent dye Calcein release from *S. aureus* CMCC26003 induced by CATH 2 and MAP34-B. Data are shown as the mean ± SEM value of three independent experiments. *** *p* < 0.001 significantly different compared to control group.

CATH 2 and MAP34-B were found to induce a concentration-dependent permeation of the cytoplasmic membrane in both *E. coli* CMCC44102 and *S. aureus* CMCC26003 (Figure 3A,B). At 5 × MIC concentrations, the permeability of CATH 2 for *E. coli* CMCC44102 was 90.00%, while for *S. aureus* CMCC26003, it was 21.36%. Similarly, the permeability of MAP34-B for *E. coli* CMCC44102 was 28.10%, whereas for *S. aureus* CMCC26003 it was 34.17%. Interestingly, CATH 2 exhibited greater permeability towards *E. coli* CMCC44102 compared to *S. aureus* CMCC26003, suggesting potential differences in their mechanisms of action.

Meanwhile, the aforementioned results were corroborated by the calcein-AM penetration test, depicted in Figure 3C. The outcomes aligned with those obtained from the PI penetration test, demonstrating that the impact of MAP34-B on *S. aureus* CMCC26003 was considerably greater compared to that of CATH 2.

### 3.5. Detection of Bacterial Outer Membrane Permeability

**Figure 4:**
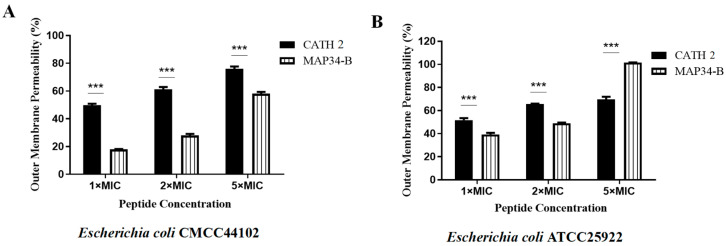
Outer membrane permeabilization assay of CATH 2 and MAP34-B. (**A**) Outer membrane permeabilization assay of CATH 2 and MAP34-B for *E. coli* CMCC44102. (**B**) Outer membrane permeabilization assay of CATH 2 and MAP34-B for *E. coli* ATCC25922. Data are shown as the mean ± SEM value of three independent experiments. *** *p* < 0.001 significantly different compared to control group.

CATH 2 and MAP34-B increased the permeability of the outer membrane of *E. coli* CMCC44102 and *E. coli* ATCC25922 in a concentration-dependent manner (Figure 4). At a concentration of 5 × MIC, the permeability of CATH 2 was 76.03% for *E. coli* CMCC44102 and 101.58% for *E. coli* ATCC25922. For MAP34-B, the permeability was 58.04% for *E. coli* CMCC44102 and 69.76% for *E. coli* ATCC25922. Notably, MAP34-B induced higher permeability in *E. coli* ATCC25922 compared to the positive control polymyxin B.

### 3.6. Anti-Inflammatory Activity 

**Figure 5:**
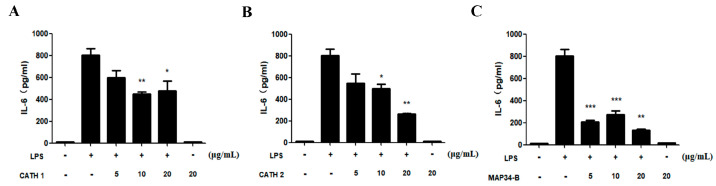
Effect of CATH 1, CATH 2, and MAP34-B on the production of pro-inflammatory cytokine IL-6 induced by LPS in mouse peritoneal macrophages. (**A**) Effect of CATH 1, (**B**) Effect of CATH 2, (**C**) Effect of MAP34-B. Data are shown as the mean ± SEM value of three independent experiments. * *p* < 0.05, ** *p* < 0.01, *** *p* < 0.001 significantly different compared to control group.

As depicted in Figure 5, the stimulation of mouse peritoneal macrophages with LPS resulted in a notable increase in the protein level of IL-6, a pro-inflammatory factor, in the cell supernatant. However, the addition of AMP samples significantly reduced this increase and showed a dose-dependent inhibition. At a concentration of 20 μg/mL, the level of IL-6 exhibited a significant decrease compared to the LPS-only group, which indicates that all three AMPs (CATH1, CATH2, and MAP34-B) exhibited potential anti-inflammatory activity.

### 3.7. Detection of Intracellular ROS

**Figure 6:**
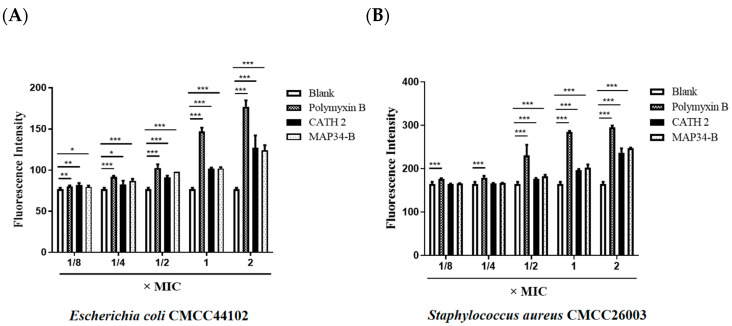
Detection of intracellular ROS. (**A**) Determination of intracellular ROS level in *E. coli* CMCC44102 induced by CATH 2 and MAP34-B. (**B**) Determination of intracellular ROS level in *S. aureus* CMCC26003 induced by CATH 2 and MAP34-B. Data are shown as the mean ± SEM value of three independent experiments. * *p* < 0.05, ** *p* < 0.01, *** *p* < 0.001 significantly different compared to control group.

The destructive effects of ROS on DNA and proteins have been well documented [37,38]. In Figure 6A,B, the results of CATH 2- and MAP34-B-induced intracellular ROS concentration in *E. coli* CMCC44102 and *S. aureus* CMCC26003 are presented. The production of ROS showed a positive correlation with the increases in CATH 2 and MAP34-B, reaching levels comparable to the positive control polymyxin B at concentrations of 1/4 and 1/8 MIC. These findings suggest that the induction of intracellular ROS production may also contribute to the bactericidal mechanisms of CATH 2 and MAP34-B.

### 3.8. DNA Binding Assay

**Figure 7:**
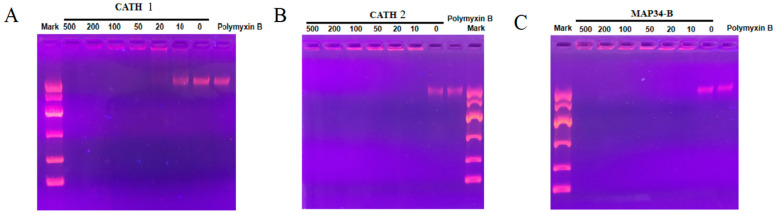
Results of the binding of AMPs with bacterial DNA. (**A**) The results of binding of CATH 1, CATH 2, and MAP34-B with bacterial DNA. (**B**) The results of binding of CATH 2 with bacterial DNA. (**C**) The results of binding of MAP34-B with bacterial DNA.

According to Figure 7A–C, the electrophoretic bands of *E. coli* genomic DNA were observed to disappear after incubation with the AMPs CATH 1, CATH 2, and MAP34-B. This indicates that the AMPs were capable of binding to *E. coli* genomic DNA, thereby preventing it from entering the agar during agarose electrophoresis. Additionally, the binding ability of CATH 2 and MAP34-B was found to be higher than that of CATH 1. These findings suggest that the three peptides may also act by binding to bacterial genomic DNA and inhibiting DNA replication and translation.

### 3.9. Antioxidant Activity

**Figure 8:**
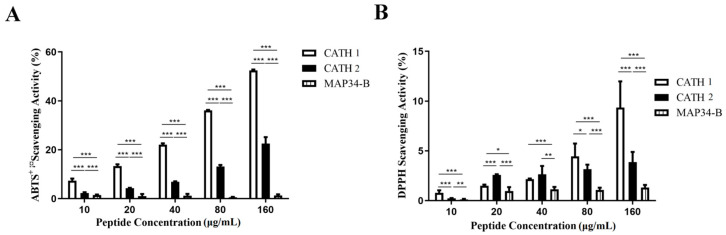
Detection of antioxidant activity of AMPs. (**A**) ABTS^+^ free radical scavenging activity of CATH 1, CATH 2 and MAP34-B. (**B**) DPPH free radical scavenging activity of CATH 1, CATH 2 and MAP34-B. Data are shown as the mean ± SEM value of three independent experiments. * *p* < 0.05, ** *p* < 0.01, *** *p* < 0.001 significantly different compared to control group.

The antioxidant activity of AMPs was assessed using the ABTS·^+^ and DPPH methods. As shown in Figure 8, there was a positive correlation between the scavenging ability of CATH 1 and CATH 2 towards ABTS·^+^ and DPPH radicals with increasing concentration. At a concentration of 160 μg/mL, CATH 1 demonstrated scavenging rates of 52.46% for ABTS·^+^ and 9.35% for DPPH radicals, while CATH 2 demonstrated rates of 22.51% for ABTS·^+^ and 3.86% for DPPH radicals. Both CATH 1 and CATH 2 demonstrated significant antioxidant activity. In contrast, MAP34-B showed no detectable antioxidant activity within the tested concentration range (10–160 μg/mL), with scavenging rates lower than 1.5%.

### 3.10. Cytotoxicity and Hemolysis Analysis

**Figure 9:**
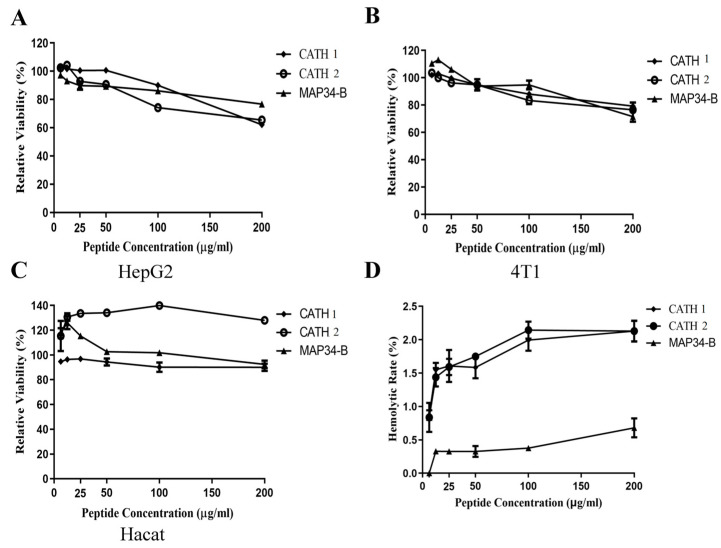
Cytotoxicity and hemolysis analysis of CATH 1, CATH 2, and MAP34-B. (**A**) Toxicity of antimicrobial peptides to HepG2 cells. (**B**) Toxicity of antimicrobial peptides to 4T1 cells. (**C**) Toxicity of antimicrobial peptides to hacat cells. (**D**) Results of hemolysis test of CATH 1, CATH 2, and MAP34-B.

The cytotoxicity of CATH 1, CATH 2, and MAP34-B against HepG2, 4T1, and hacat cells was found to be lower than 40% at a concentration of 200 µg/mL (Figure 9A–C). Therefore, it can be concluded that the cytotoxicity of CATH 1, CATH 2, and MAP34-B was weak.

The hemolytic activities of CATH 1, CATH 2, and MAP34-B were also evaluated and the results are shown in Figure 9D. At a concentration of 200 µg/mL, the hemolytic activities of CATH 1, CATH 2, and MAP34-B were 2.13%, 2.13% and 0.68%, respectively. These findings indicate that CATH 1, CATH 2, and MAP34-B exhibited minimal hemolytic activity.

## 4. Discussion

AMPs are small molecular mass proteins that exhibit antimicrobial, antiviral, and antifungal activities. They are an essential part of the intrinsic immune response in various organisms including plants, animals, and humans. AMPs are also referred to as host-defense peptides [39,40]. These peptides display a high level of structural and functional diversity both within and across species. Numerous AMPs have been isolated from vertebrates such as cattle, pigs, sheep, goats, mice, and rats [24,40,41,42,43]. Due to their potent antimicrobial activity and minimal resistance development, AMPs are considered promising candidates for the development of new antimicrobial drugs. Currently, over 30 AMP-related drugs are undergoing clinical studies [44]. 

There have been limited studies on AMPs in goats. Only a few AMPs, such as Bac5 (cathelicidin 2, CATH 2), BAC 7.5 (cathelicidin 3), MAP 28 (cathelicidin 6), MAP 34 A and B (cathelicidin 7) and ChBac 3.4 have been reported. Shamova et al. discovered two AMPs, minibactenecins ChBac7.Nα and ChBac7.Nβ with strong antimicrobial activity in goat neutrophils. AMPs show promising medicinal potential due to their effective antimicrobial properties against Gram-negative bacteria, Gram-positive bacteria, and certain drug-resistant strains. Additionally, some of them exhibit no hemolytic effect on human red blood cells and no cytotoxicity, further enhancing their therapeutic value [27]. Bac 5, isolated from goat neutrophils, exhibits the activity to bind to LPS and eliminate various Gram-negative bacteria, including *E. coli* ML-35. This activity is observed at NaCl concentrations similar to that found in the extracellular fluid, suggesting its potential role in defending against Gram-negative bacterial infections in ruminants [24]. Additionally, Srisaikham et al. discovered the secretion of Bac 5 by goat white blood cells even without LPS stimulation [45]. ChBac3.4, a proline-rich polypeptide isolated from goat leukocytes, exhibits more than 50% sequence identity with Bac 5 and demonstrates broad-spectrum antimicrobial activity in low-salinity environments. In vitro studies have shown that ChBac 3.4 exhibits selective cytotoxicity [6]. MAP34 (Cath-7), which is expressed in goat mammary leukocytes and secreted in milk, has been found to inhibit IL-6 expression in white blood cells when combined with LPS [46]. The expression of MAP28 and MAP34 varies depending on the environmental factors and is observed in the mammary gland of goats. In lentivirus (SRLV)-infected goats, the expression of the MAP28 gene is elevated in somatic milk cells (MSCs) compared to blood leukocytes. Additionally, the concentration of MAP28 in milk surpasses that found in the bloodstream. Conversely, SRLV-infected goats exhibit reduced levels of MAP28 and increased levels of MAP34 in their milk when compared to uninfected goats [47]. Furthermore, the expression of BAC7.5 in mammary gland cells increases upon the addition of selenized yeast (Se-yeast) to the diet [48]. These findings highlight the role of AMPs in host immunity. The study demonstrated that the synergistic combination of MAP-28 and Mini-ChBac7.5Nα not only increased antimicrobial activity but also minimized the risk of resistance development, particularly at lower therapeutic doses. Additionally, the combination of ChMAP-28 and Mini-ChBac7.5Nα showed enhanced antimicrobial activity and reduced the likelihood of drug resistance at lower therapeutic doses [49]. These results emphasize the significance of having multiple AMPs present in the same tissue.

In our previous transcriptome analysis of goat submandibular glands, we identified three cationic AMPs that were highly expressed in goat kids: CATH 1, CATH 2, and MAP34-B. However, the activity study of CATH 2 only focused on antimicrobial activity, while the activities of CATH 1 and MAP34-B were not reported. Therefore, this study aims to further investigate the structure, function, and antimicrobial mechanism of these three AMPs. 

The process of cationic AMP-mediated sterilization can generally be divided into several steps. Firstly, AMPs are attracted to the surfaces of bacteria through electrostatic bonding between cationic polypeptides and anionic substances on the bacterial surface, such as anionic phospholipids, lipopolysaccharides in Gram-negative bacteria, and lipophosphorus acids in Gram-positive bacteria [39]. Once close to the bacterial surface, these peptides can interact with the lipid bilayer, leading to the adsorption and embedding of the lipid head groups. Among all the strains tested, MAP34-B exhibited antimicrobial activity against all Gram-negative bacteria, while only some Gram-positive bacteria were affected. This is similar to the reported pig cathelicidin, PMAP-37 [50]. CATH 2 demonstrated similar antimicrobial activity to MAP34-B, albeit slightly lower (Table 1). This above observation highlights the broad-spectrum antimicrobial activity of AMPs derived from goat submandibular glands, which aligns with the diverse oral feeding environments of goats. In the predicted structure of MAP34-B, two α-helices were formed at the N terminal and the C terminal, respectively. However, only a portion of the amino acid residues in CATH 1 and CATH 2 formed α-helices, while the remaining residues were randomly curled (Figure 1). As reported by Bolosov et al., chDode, homolog of CATH1, formed a β-structural antiparallel covalent dimer, stabilized by two intermonomer disulfide bonds [51]. This observation deviated from our initial prediction for CATH1 monomer. We chemically synthesized the intramolecular disulfide bonds for CATH1, which may have contributed to the lack of CATH1 activity detected. 

Unlike many AMPs, proline-rich AMPs inactivate Gram-negative bacteria through a non-lysis mechanism. Several pieces of evidence indicate that AMPs are internalized into bacteria and regulate their activity by interacting with unknown cellular components [52]. Bactericidal kinetics elucidate the antibacterial or bactericidal mechanism of an antibacterial agent, as well as its prompt onset of action. The mechanism of action that directly eradicates bacteria would render them less susceptible to persistent peptide resistance. This is particularly advantageous in the current context of increasing drug-resistant bacterial strains. Although both MAP34-B and CATH 2 have the same MIC against *E. coli* and *S. aureus*, CATH 2 exhibits a significantly higher bactericidal rate against both bacteria compared to MAP34-B. The reason for this could be attributed to the higher concentration of cation in CATH 2. Compared to ampicillin, both CATH 2 and MAP34-B exhibited significantly higher bactericidal rates against *S. aureus*. In the case of *E. coli*, CATH 2 demonstrated significantly higher bactericidal rates compared to ampicillin, while MAP34-B showed comparable results. The results suggest that CATH 2 exhibits a superior antibacterial efficacy compared to MAP34-B. Previous studies have indicated that most AMPs primarily target the bacterial cytoplasmic membrane [53]. These AMPs exert microbicidal activity by selectively targeting and disrupting bacterial plasma membrane structures, primarily through osmosis-induced cellular leakage of contents. To investigate the bactericidal mechanism of CATH 2 and MAP34-B, we conducted cytoplasmic membrane permeability experiments. The findings revealed that the permeation of the plasma membrane by *S. aureus* and *E. coli* was dependent on the dosage, and CATH 2 and MAP34-B exhibited distinct effects (Figure 3). The results imply that the bactericidal process initiates with the accumulation and aggregation of AMP molecules, facilitating efficient penetration via interaction between positively charged residues of antimicrobial peptides and the negatively charged regions on the bacterial membrane surface. It is reasonable to assume that CATH 2 and MAP34-B exert their functions by entering the cytoplasm, and the changes in cytoplasmic permeability could reflect the transmembrane transport of peptides. The outer membrane of Gram-negative bacteria possesses a unique structure that acts as a barrier against the permeation of antibiotics and other harmful chemicals [54]. CATH 2 and MAP34-B also demonstrated a dose-dependent penetration of the *E. coli* outer membrane (Figure 4). Furthermore, these peptides were found to significantly increase the production of ROS in bacterial cells, suggesting that this may be one of their antibacterial mechanisms (Figure 6). Another mechanism of action for antimicrobial peptides is their ability to inhibit nucleic acid synthesis through non-targeted membrane mechanisms. DNA binding revealed that CATH 1, CATH 2, and MAP34-B directly bind to *E. coli* DNA possibly through charge attraction (Figure 7). This finding implies that these three AMPs bind to the bacterial DNA upon entering the cells, thereby inhibiting bacterial DNA replication and transcription [55]. In addition to their broad-spectrum antibacterial activity, certain AMPs have been found to play a crucial role in host immune responses against pathogenic infections [56]. Notably, CATH 1, CATH 2, and MAP34-B exhibit potential anti-inflammatory activity and significantly suppress the production of the pro-inflammatory cytokine IL-6 induced by LPS in macrophages. Additionally, CATH 1 and CATH 2 exhibit significant antioxidant activity (Figure 8), unlike MAP34-B, indicating their capacity to counteract damage induced by ROS through chemical reactions, whether expressed intracellularly or secreted extracellularly. This underscores the role of antioxidant activity in CATH 1 and CATH 2 as a self-defense mechanism of the host. Therefore, CATH 1, CATH 2, and MAP34-B have the potential to participate in the oral immune response to pathogen infection in goats, acting as host defense. 

ROS production was positively correlated with the increased concentrations of CATH 2 and MAP34-B, indicating that the induction of intracellular ROS production could be one of the bactericidal mechanisms of CATH 2 and MAP34-B. Upon bacterial infection in the host, AMPs disrupt the integrity of the bacterial cell membrane, potentially impeding or modulating the electrical transmission chain on membrane. Consequently, this disruption induces ROS production and subsequently enhances bactericidal activity. The antioxidant activity of AMPs primarily serves to protect the host, while the induction of ROS production takes place within the bacterial cell. Therefore, these two functions are not contradictory but rather complementary in their roles. Furthermore, these peptides showed minimal cytotoxicity to mammalian cells, including tumor and normal cell lines (Figure 9). Considering all these characteristics, CATH 2 and MAP34-B demonstrate promising therapeutic potential for treating microbial infections. 

The AMPs CATH 2 and MAP34-B, derived from the goat submandibular gland, have demonstrated antibacterial activity by targeting the cell membrane. Additionally, they have shown strong anti-inflammatory activity by effectively inhibiting the production of pro-inflammatory cytokine IL-6 in mouse macrophages stimulated with LPS. CATH1, on the other hand, exhibited significant antioxidant activity but did not display antimicrobial activity against the tested strains. The versatile functions of CATH 1, CATH 2, and MAP34-B highlight their crucial roles in the host’s immune defense against pathogen invasion and external environmental stress. Their potent antibacterial, anti-inflammatory, and antioxidant properties, along with their low cytotoxicity to mammalian cells, offer valuable insights for further research on their immunological role in host protection.

## Figures and Tables

**Table 1:** Physical and chemical properties of antimicrobial peptides.

Name	Sequence	Number of Amino Acids	pI	Molecular Weight (Da)	
				Theoretical	Measured
MAP34-B	GLFGRLRDSLRRGGQKILEKVERIGDRIKDIFRG	34	11.29	3955.63	3955.66
CATH 1	RITKQPWAPPQAARICQFVLIRVCR	25	11.54	2949.59	2949.61
CATH 2	RFRLPFRRPPIRIHPPPFYPPFRRFLGRR	29	12.54	3746.53	3746.56

**Table 2:** MIC results of AMPs.

Microorganisms	MIC (µg/mL)				
	CATH 1	CATH 2	MAP34-B	Ampicillin	Meropenem
Gram-negative bacteria					
*Escherichia coli* CMCC44102	>100	37.5	18.75	2.34	1.17
*Escherichia coli* ATCC25922	>100	37.5	37.5	2.34	0.02
*Pseudomonas aeruginosa* CMCC10104	>100	75	75	>100	0.04
*Shigella castellani* ATCC12022	>100	75	37.5	1.17	0.08
*Acinetobacter baumnnii* ATCC19606	>100	37.5	18.75	>100	0.04
Gram-positive bacteria					
*Staphylococcus aureus* CMCC26003	>100	18.75	18.75	0.59	0.15
*Staphylococcus aureus* ATCC25923	>100	>100	>100	0.59	0.15
*Clostridium perfringens* ATCC13124	>100	37.5	9.38	2.34	0.02
*Enterococcus faecalis* ATCC29212	>100	>100	>100	0.59	0.59
Fungus					
*Candida albicans* CMCC98001	>100	>100	>100	>100	>100

## Data Availability

The data presented in this study are available in this article.
